# Time-Dependent Electrochemical Behavior of PVA/Chitosan Anti-Corrosion Coatings on Biomedical-Grade Steel in Simulated Physiological Conditions

**DOI:** 10.3390/jfb17080363

**Published:** 2026-07-28

**Authors:** Antonio V. Vega, Arnold Solano, Fausto Acosta-Fiallos, Raúl Dávalos Monteiro

**Affiliations:** 1 School of Biological Sciences and Engineering, Yachay Tech University, Urcuquí 100119, Ecuador; antonio.vega@yachaytech.edu.ec (A.V.V.); arnold.solano@yachaytech.edu.ec (A.S.); 2 Facultad de Ingeniería en Mecánica y Ciencias de la Producción, Escuela Superior Politécnica del Litoral (ESPOL), Guayaquil 90902, Ecuador; fausacos@espol.edu.ec

**Keywords:** PVA/chitosan coatings, electrochemical impedance spectroscopy, corrosion protection, biomedical steel, biopolymer coatings, diffusion mechanisms, surface engineering, physiological conditions

## Abstract

The corrosion of metallic biomaterials under physiological conditions remains a critical challenge due to the risk of ion release affecting biocompatibility. In this study, the time-dependent electrochemical behavior of a poly(vinyl alcohol) (PVA)/chitosan biopolymer coating applied to biomedical-grade steel was evaluated to assess its protective performance. The coating was prepared via dip-coating using a 9.9:0.1 PVA/chitosan formulation and characterized by FTIR and Raman spectroscopy to confirm intermolecular interactions and film formation. Electrochemical impedance spectroscopy was employed over 216 h of immersion in Hanks’ Balanced Salt Solution to monitor coating degradation and corrosion mechanisms. The coated system exhibited a significant increase in the low-frequency impedance modulus from approximately 1200–1400 to 5000–6000 Ω·cm^2^, indicating enhanced barrier properties and interfacial stability compared to uncoated steel. Equivalent circuit analysis revealed a transition from a predominantly capacitive, barrier-controlled response at early immersion stages to diffusion-influenced behavior at longer exposure times, associated with electrolyte penetration and interfacial processes. Overall, the PVA/chitosan coating effectively delays corrosion reactions and maintains high impedance under simulated physiological conditions, demonstrating its potential as a protective and biocompatible surface modification for metallic biomedical applications.

## 1. Introduction

Metallic biomaterials are widely used in orthopedic, dental, and surgical applications due to their good mechanical properties, durability, and relatively high corrosion resistance [1,2,3,4]. Among them, biomedical-grade steels remain one of the most widely used materials because of their favorable combination of mechanical performance and cost-efficient values. However, despite the latter advantages, metallic biomedical materials are naturally susceptible to degradation when exposed to physiological environments, which are typically rich in chloride ions, biological agents such as proteins, micro-environments with pH shifts, and dissolved oxygen at low concentrations [5,6]. These conditions can promote localized corrosion processes, leading to the release of metallic ions that may induce adverse biological responses and compromise long-term implant performance [7,8].

In addition to electrochemical degradation (corrosion), metallic biomaterials are also susceptible to surface-related issues, including poor osteointegration [9,10] and the formation of biofilms by pathogenic microorganisms [11]. These phenomena can produce inflammatory responses and increase the risk of future implant failure. As a result, some research efforts have been directed toward modifying the surface properties of metallic substrates to improve their corrosion resistance and, therefore, their biological performance [5,12,13]. Among the available strategies, the application of protective coatings has emerged as an effective approach to act as a barrier against aggressive species while simultaneously improving biocompatibility [14,15,16,17,18].

Polymeric coatings have been widely investigated as environmentally friendly alternatives for corrosion protection of metallic substrates, particularly in replacing traditional chromate-based systems. Among these, biopolymers have gained increasing attention due to their biodegradability, low toxicity, and film-forming ability [19,20]. Biopolymeric systems have demonstrated enhanced corrosion protection performance due to the formation of hydrogen-bonded networks that reduce permeability and improve coating integrity [21,22].

In addition to polymer-based coatings, a wide range of surface modification strategies have been developed for biomedical applications. Among these, ceramic coatings such as hydroxyapatite (HA) have been extensively used due to their excellent bioactivity and ability to promote osseointegration, making them suitable for orthopedic and dental implants [23]. Similarly, zirconium dioxide (zirconia) coatings exhibit high mechanical strength, chemical stability, and wear resistance, which are advantageous for load-bearing applications [24]. Inorganic coatings, including carbon-based films such as diamond-like carbon (DLC), as well as nitride coatings like titanium nitride (TiN), have also been widely investigated. These coatings provide improved hardness, corrosion resistance, and reduced friction; however, they often require complex deposition techniques and may suffer from adhesion issues [25,26]. Silicon-based coatings and their derivatives have also shown promise due to their chemical stability and tunable surface properties [27]. Despite these advances, many inorganic coatings lack intrinsic biological functionality [19].

In this regard, biopolymer-based coatings have attracted attention due to their low toxicity, biodegradability, and potential for functionalization. Chitosan, a naturally derived polysaccharide, exhibits excellent biocompatibility, antibacterial properties, and film-forming ability. Its cationic nature allows strong interactions with negatively charged bacterial cell membranes, providing antimicrobial functionality, while its ability to form adherent films on metallic substrates makes it a promising corrosion barrier. On the other hand, poly(vinyl alcohol) (PVA) is a semicrystalline polymer known for its good mechanical properties, chemical stability, and compatibility with biological systems. Previous studies have shown that combining PVA with other polymers can enhance coating performance, particularly in terms of corrosion resistance and material integrity.

A comparative summary of commonly used biomedical coatings is presented in Table 1, highlighting the advantages and limitations of each system relative to the proposed PVA/chitosan coating.

Even though, these promising characteristics, there is still a limited understanding of the time-dependent electrochemical behavior of PVA/chitosan coatings under simulated physiological conditions. In particular, the mechanisms governing the degradation of such coatings and their ability to provide sustained corrosion protection over time remain insufficiently explored. Electrochemical impedance spectroscopy (EIS) is a powerful and non-destructive technique that enables the investigation of interfacial processes and coating performance by analyzing the response of a system to small perturbations over a wide frequency range [28,29,30]. This technique is especially suitable for monitoring coating degradation and corrosion mechanisms in situ [31,32].

Therefore, the aim of this study is to evaluate the corrosion performance of a hybrid polyvinyl alcohol/chitosan biopolymer coating applied directly to commercially available surgical-grade steel substrates under simulated physiological conditions using electrochemical impedance spectroscopy. Particular emphasis is placed on analyzing the temporal evolution of the electrochemical response and identifying the dominant mechanisms governing coating degradation and corrosion processes. The novelty of this work lies in the integration of a low-cost, biocompatible, and easily processable polymeric system with a systematic electrochemical assessment on real surgical materials. Unlike conventional ceramic or nitride coatings, the proposed PVA/chitosan coating offers additional advantages such as potential antibacterial functionality and straightforward application, highlighting its potential as an effective protective strategy for metallic biomaterials.

## 2. Materials and Methods

### 2.1. Steel Grade Characterization

The metallic substrate used in this study consisted of commercially available medical scalpel blades employed as received, without prior specification of their chemical composition by the manufacturer. Therefore, X-ray diffraction analysis was used to identify the crystalline structure of the material. The diffraction pattern revealed characteristic peaks of ferritic or low-alloy steel. The absence of additional diffraction peaks associated with chromium-rich phases or austenitic phases suggests that the substrate does not correspond to a typical austenitic stainless steel. This result is consistent with the use of cost-effective steel substrates in disposable surgical blades and supports its validity as a representative model for evaluating corrosion behavior and protective coating performance.

### 2.2. Metal Surface Preparation

Medical-grade scalpel blades were cut into samples with an exposed area of 1 cm^2^. The samples were mechanically polished using a polishing system equipped with a number 4000 diamond grit disk at 600 rpm under continuous oil-based lubrication to ensure a uniform surface finish. Polishing was performed in orthogonal directions to minimize surface irregularities.

Following the polishing process, samples underwent ultrasonic cleaning in 98 wt% ethanol for 5 min to remove particulate debris. To eliminate residual organic contaminants, the samples were immersed in acetone for 2 min. Finally, they were rinsed with distilled water, immediately dried using a pressurized nitrogen stream at room temperature, and stored for subsequent coating application.

### 2.3. Coating Formulation

A 12% (*w*/*v*) PVA solution was prepared by dissolving PVA powder (Sigma-Aldrich, St. Louis, MO, USA) (Mw = 30,000 g/mol) in ultrapure water at 70 °C under constant stirring until complete dissolution, followed by cooling to room temperature. A 1.7% (*w*/*v*) chitosan solution was prepared by dissolving high molecular weight chitosan (85% deacetylation, Sigma-Aldrich, USA) (in a 1% (*v*/*v*) acetic acid aqueous solution under constant stirring at 50 °C for 4 h. The PVA/chitosan coating was formulated by mixing both solutions in a volumetric ratio of 9.9:0.1 under continuous stirring at 60 °C for 2 h, followed by cooling to room temperature prior to application.

### 2.4. Coating Application

The PVA/chitosan coating was deposited onto the polished metal substrates using a dip-coating technique. The substrates were vertically immersed into the coating solution and held for a dwell time of 2 min to ensure complete surface wetting and film formation. Subsequently, the samples were withdrawn at a constant speed to promote a uniform and reproducible layer. The coated substrates were then allowed to dry under ambient conditions for 2 h. This method was selected to enhance thickness homogeneity and ensure a continuous protective barrier across the exposed surface.

### 2.5. X-Ray Diffraction

XRD analysis was performed to identify the crystalline phases present in the metallic substrate. Measurements were performed using Cu Kα radiation (λ=1.5406 Å) over a 2θ range from $10^{\circ }$ to $100^{\circ }$, with a step size of $0.01^{\circ }$. The X-ray source was operated at 40 kV and 15 mA. Data were collected in θ–2θ configuration to obtain diffraction patterns representative of the bulk crystalline structure of the metallic samples.

### 2.6. Fourier Transform Infrared Spectroscopy

Fourier transform infrared (FTIR) spectroscopy was performed to identify the functional groups present in the polymeric coating. The analysis was carried out using an attenuated total reflectance (ATR) module. Spectra were recorded over a wavenumber range of 650 to 4000 cm^−1^ with a resolution of 4 cm^−1^, using eight scans per sample.

### 2.7. Raman Spectroscopy

Raman spectroscopy was performed to analyze the molecular structure and vibrational modes of the polymeric coatings. Measurements were carried out using a 532 nm excitation laser and a 100× objective lens. Spectra were recorded over a range of 500 to 3250 cm^−1^ with an acquisition time of 15 s and 15 accumulations per measurement to improve the signal-to-noise ratio.

Raman analysis was used to complement FTIR results and confirm the presence of characteristic functional groups of both PVA and chitosan in the coating.

### 2.8. Electrochemical Testing

#### 2.8.1. Sample Preparation

For electrochemical measurements, the metallic samples were electrically connected using a copper wire attached to the substrate with conductive solder. The non-working areas were insulated using a protective varnish to ensure that only the defined surface was exposed to the electrolyte. Samples were divided into two groups: coated samples (treatment) and uncoated samples (control).

#### 2.8.2. Electrolyte Solution

Electrochemical experiments were conducted in Hanks’ Balanced Salt Solution (HBSS) to simulate physiological conditions. The solution was prepared using analytical-grade reagents, including NaCl, KCl, CaCl_2_, NaHCO_3_, KH_2_PO_4_, Na_2_HPO_4_·2H_2_O, MgSO_4_·7H_2_O, MgCl_2_·6H_2_O, and glucose.

The composition of the Hanks’ Balanced Salt Solution (HBSS) used as electrolyte is summarized in Table 2.

This solution was selected to simulate physiological conditions, providing relevant ionic species such as chlorides, phosphates, and bicarbonates that influence the electrochemical behavior of the system. The measured pH of the solution was 7.4, and the electrolyte was maintained at 37 °C during testing.

#### 2.8.3. Electrochemical Impedance Spectroscopy

Electrochemical impedance spectroscopy (EIS) measurements were performed using a three-electrode configuration, consisting of the sample as the working electrode, a saturated Ag/AgCl reference electrode, and a platinum counter electrode.

Measurements were conducted at open circuit potential (OCP) using a sinusoidal perturbation of 10 mV amplitude over a frequency range from 100 kHz to 0.01 Hz. Data were acquired with 10 points per decade.

The electrochemical response of the samples was monitored over an immersion period of up to 9 days to evaluate the time-dependent behavior of the coating system under simulated physiological conditions.

## 3. Results

### 3.1. X-Ray Diffraction Analysis

**Figure 1:**
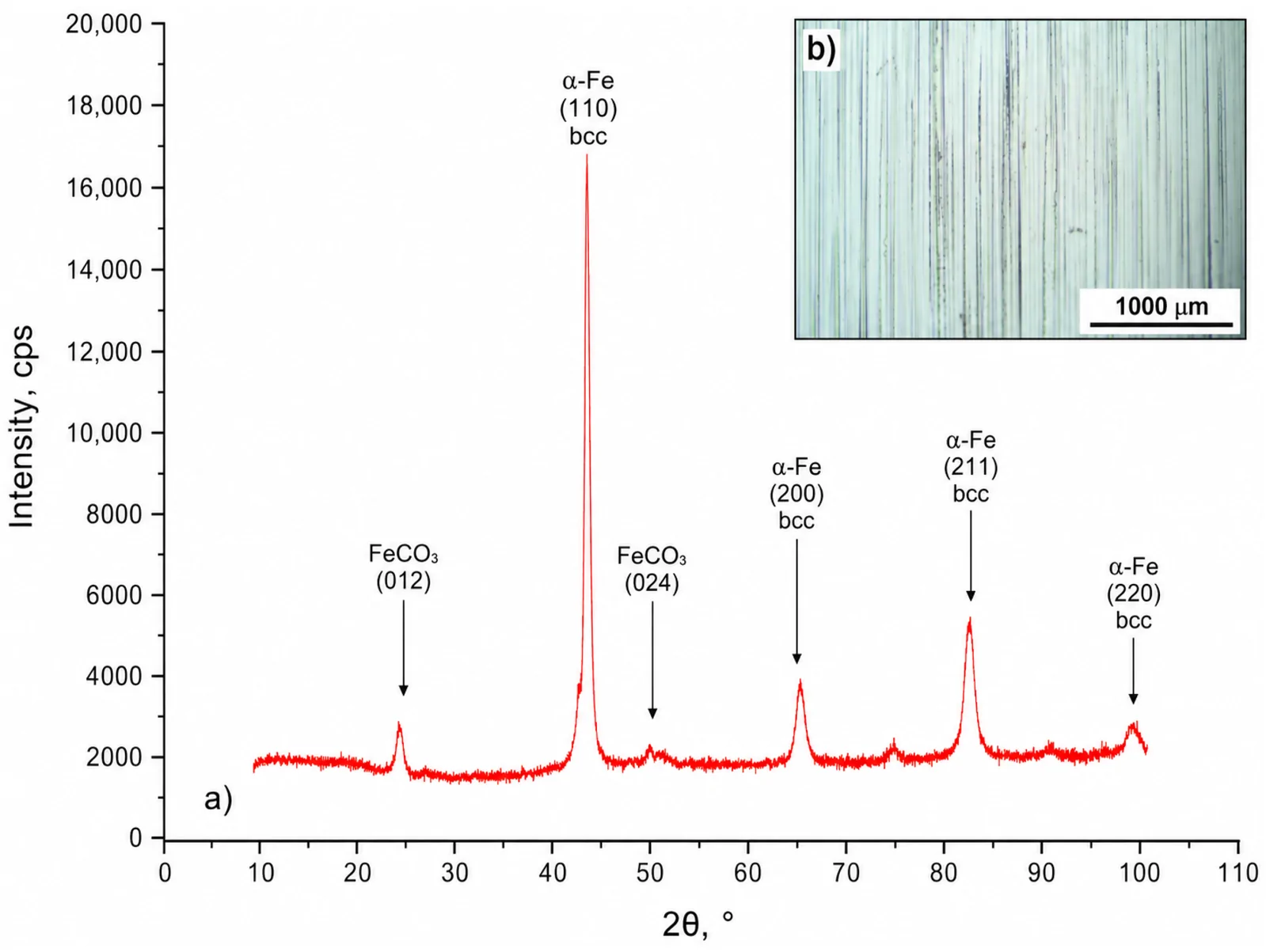
(**a**) X-ray diffraction pattern of the medical-grade steel substrate prior to electrochemical testing; (**b**) Microscopic image of polished metallic samples.

The X-ray diffraction pattern of the metallic substrate is shown in Figure 1. The main diffraction peaks observed at approximately $2\theta =44.5^{\circ }$, $64.5^{\circ }$, and $82.1^{\circ }$ correspond to the (110), (200), and (211) planes of α-ferrite, indicating a body-centered cubic (bcc) crystal structure [33]. This phase is characteristic of low-carbon steels commonly used in medical-grade cutting instruments [34].

The predominance of the ferritic phase suggests a relatively homogeneous substrate, with no significant secondary phases detected within the sensitivity of the technique. Minor low-intensity features may be associated with surface compounds or residual species formed most probably during sample handling.

The identification of a ferritic microstructure is relevant for the corrosion analysis, as such materials are known to exhibit susceptibility to localized corrosion in chloride-containing environments [35,36,37]. Therefore, the initial microstructural condition of the substrate are important to consider in the electrochemical behavior observed during immersion testing.

### 3.2. FTIR and Raman Analysis

**Figure 2:**
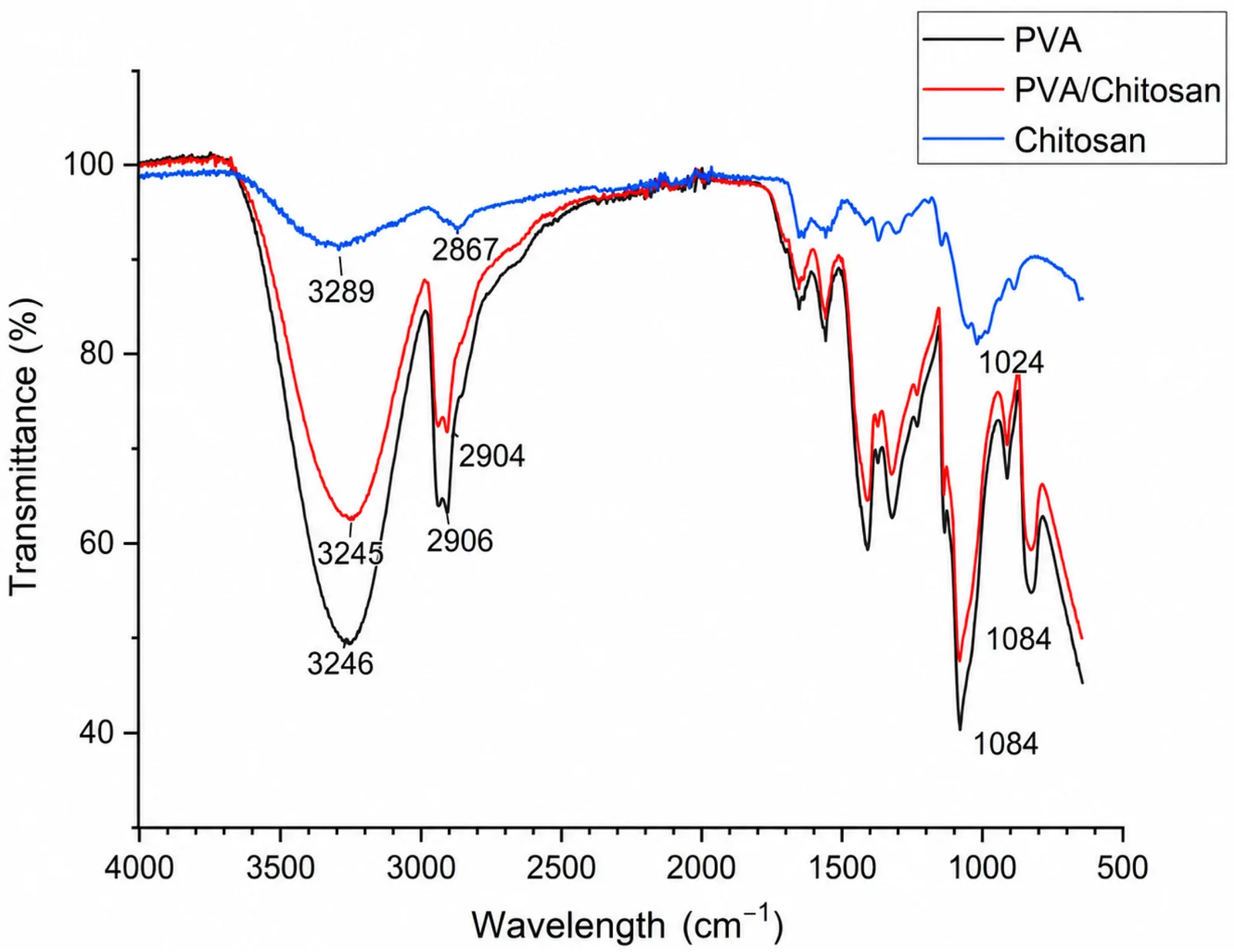
FTIR spectrum of the PVA/chitosan coating.

Figure 2 shows the FTIR spectrum of the PVA/chitosan coating. The broad absorption band observed around 3200–3500 cm^−1^ is attributed to O–H stretching vibrations, indicating the presence of hydroxyl groups from both PVA and chitosan. The peaks observed near 2900 cm^−1^ correspond to C–H stretching vibrations.

A characteristic peak around 1650 cm^−1^ can be associated with amide I groups (C=O stretching) from chitosan, while bands near 1550 cm^−1^ are related to N–H bending, confirming the presence of amino groups. Additionally, peaks in the region of 1000–1150 cm^−1^ are assigned to C–O stretching vibrations, characteristic of the polymer backbone.

**Figure 3:**
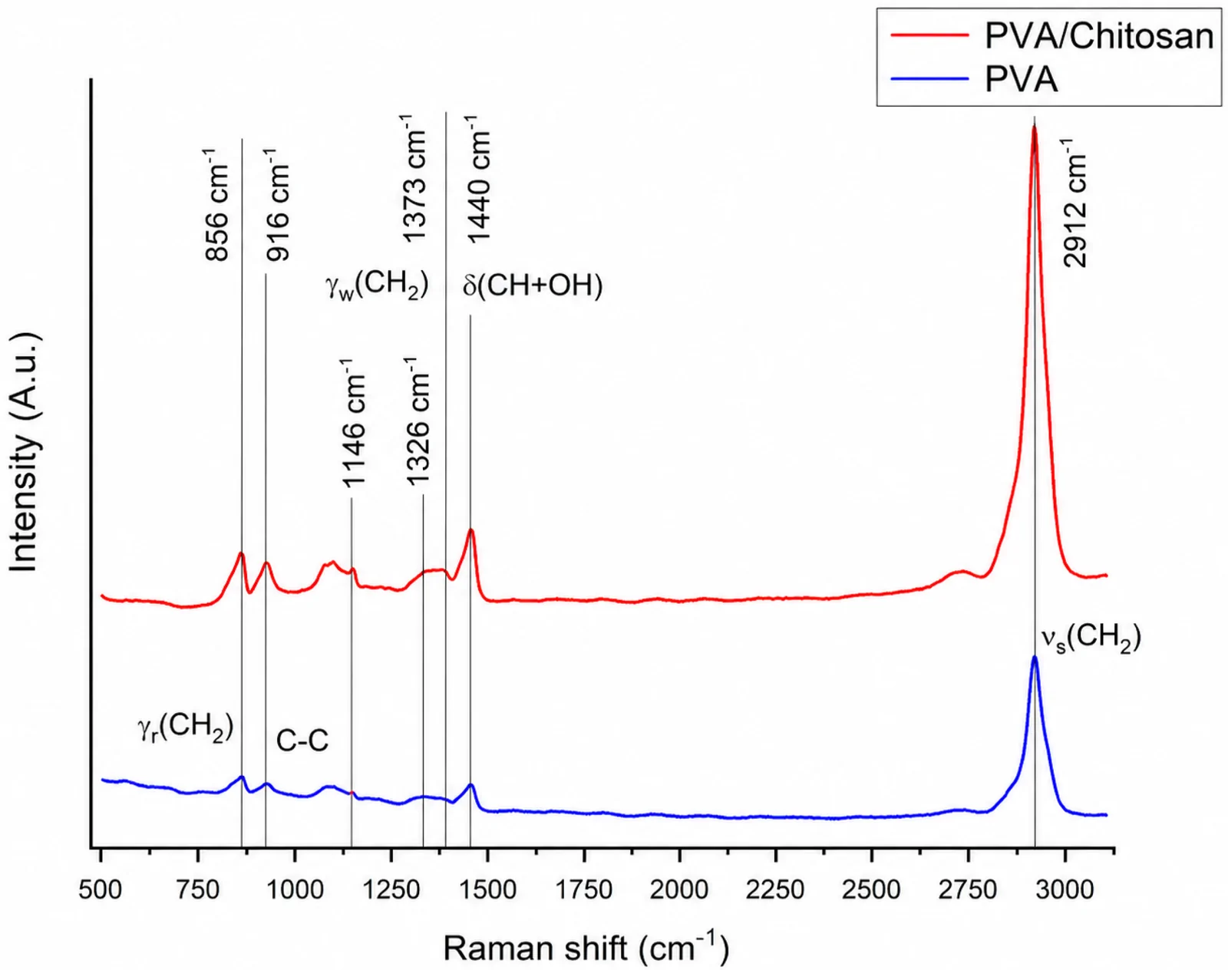
Raman spectrum of the PVA/chitosan coating.

Figure 3 presents the Raman spectrum of the coating. The observed bands are consistent with the vibrational modes of both PVA and chitosan, supporting the successful formation of the polymeric coating. The presence of overlapping features in both FTIR and Raman spectra suggests possible intermolecular interactions, such as hydrogen bonding, between PVA and chitosan chains.

These interactions may contribute to the formation of a cohesive polymer network, which is relevant for the barrier properties of the coating in corrosion protection.

### 3.3. Electrochemical Impedance Spectroscopy (EIS)

**Figure 4:**
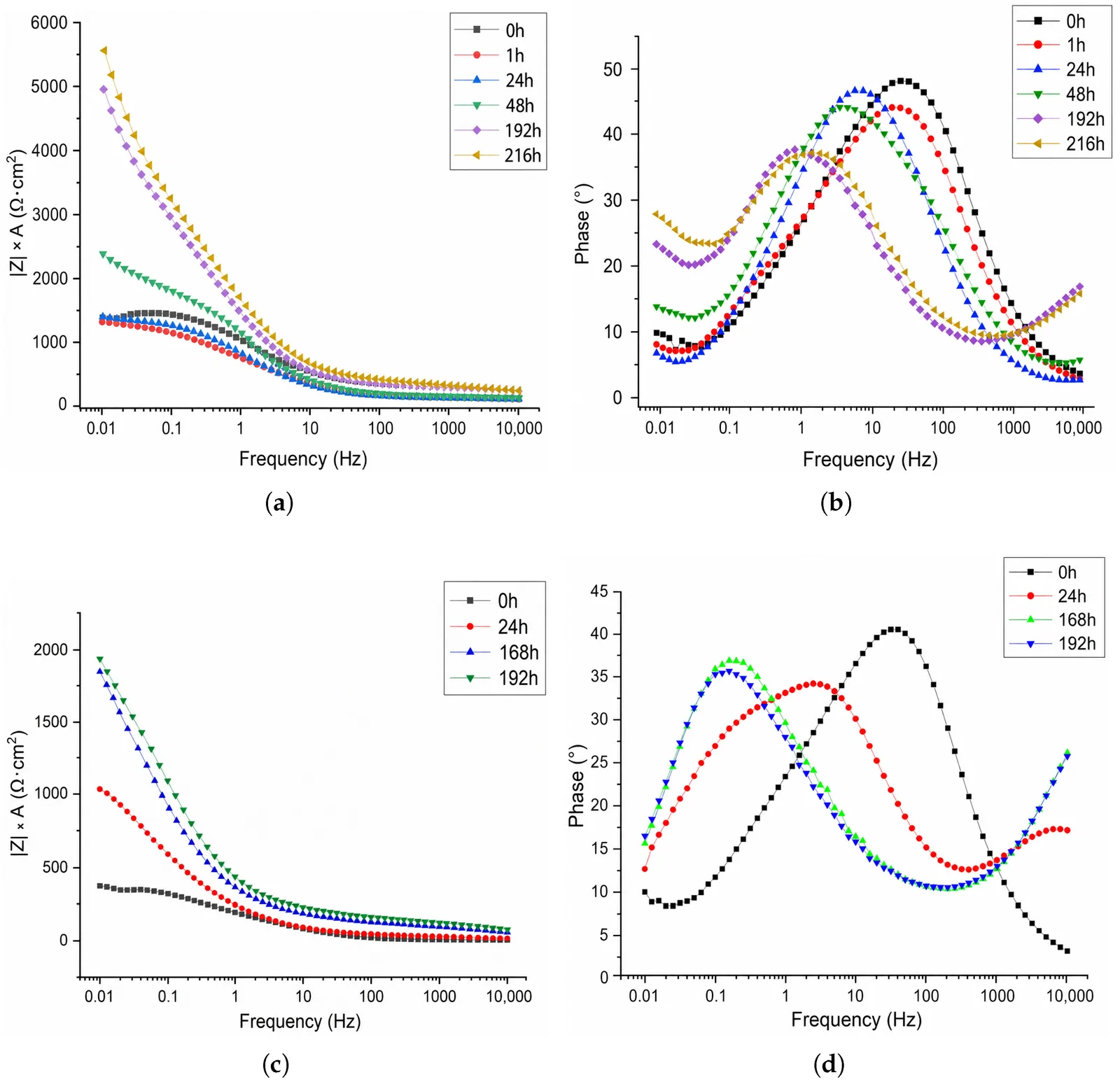
EIS spectra acquired for coated and control samples immersed in HBSS solution for 192 h: (**a**) Coated sample Impedance modulus, (**b**) Coated sample phase angle, (**c**) Control sample Impedance modulus, and (**d**) Control sample phase angle.

Figure 4 shows the Bode impedance spectra for both coated (treatment) and uncoated (control) samples during immersion in HBSS for up to 216 h.

At high frequencies (10^2^–10^4^ Hz), the impedance modulus (|Z|) and phase angle remain relatively constant for both systems, corresponding to the solution resistance ($R_{s}$). This behavior is consistent throughout the immersion period, indicating stable electrolyte conditions.

At low frequencies (0.01 Hz), the coated samples (Figure 4a) exhibit an increase in impedance modulus from approximately 1200–1400 Ωcm^2^ during the first 24 h to values in the range of 5000–6000 Ωcm^2^ after prolonged immersion. This increase suggests the formation of a protective layer, likely associated with corrosion products and/or coating restructuring, which enhances the resistance to charge transfer.

The phase angle response (Figure 4b) shows a gradual decrease over time, indicating a deviation from ideal capacitive behavior. This suggests increasing heterogeneity at the coating/metal interface, possibly due to water uptake, coating degradation, or the formation of corrosion products.

In contrast, the uncoated samples (Figure 4c) exhibit a rapid evolution in impedance behavior during the first 24 h, with noticeable changes in the mid- and low-frequency regions. The impedance increases with time, which may be attributed to the formation of corrosion product layers on the steel surface.

The phase angle plots for the uncoated system (Figure 4d) show a progressive shift in the peak towards lower frequencies, indicating slower electrochemical processes and the development of diffusion-controlled behavior. The decrease in phase angle and peak broadening further suggest the formation of non-uniform and porous corrosion layers.

Overall, the coated system exhibits higher impedance values and a more stable electrochemical response compared to the bare steel, indicating improved corrosion resistance provided by the polymeric coating.

### 3.4. Equivalent Circuit Analysis and Physical Interpretation

The electrochemical behavior of the coated and uncoated systems was further analyzed using equivalent electrical circuit models to provide information into the possible corrosion mechanisms.

**Figure 5:**
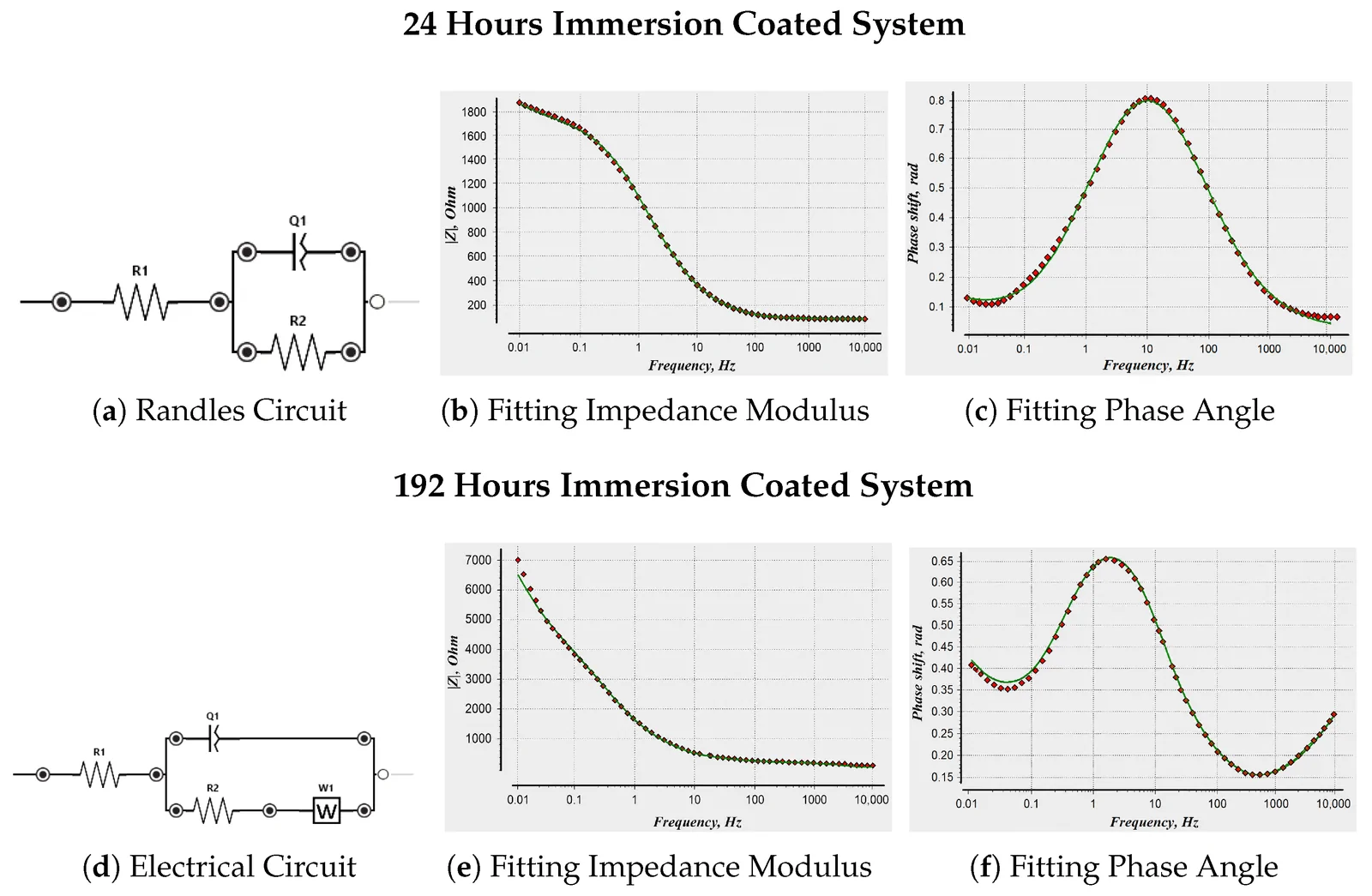
Equivalent electrical circuits used to model the electrochemical response of coated and uncoated systems at different immersion times: (**a**) Randles-type circuit describing the coating barrier behavior at early exposure (24 h), consisting of the solution resistance ($R_{1}$) in series with a parallel combination of a constant phase element (Q_1_) and coating resistance ($R_{2}$); (**d**) extended circuit including a Warburg element (*W*) to account for diffusion-controlled processes at prolonged immersion (192 h).

At early immersion times (24 h), the coated system can be described using a Randles circuit (Figure 5a), consisting of the solution resistance ($R_{s}$) in series with a parallel combination of a constant phase element ($Q_{c}$) and a coating resistance ($R_{c}$).

In this configuration, $R_{1}$ represents the electrolyte resistance, while $Q_{1}$ accounts for the non-ideal capacitive behavior of the biopolymeric coating, which is associated with surface heterogeneity and roughness. The resistance $R_{2}$ reflects the barrier properties of the coating, limiting ionic transport. The total resistance to charge transfer is associated with electrochemical reactions that occur at the metal/coating interface.

As immersion time increases (192 h), the equivalent circuit becomes more complex (Figure 5d), indicating the evolution of multiple electrochemical processes. The model incorporates an additional Warburg element (W), which indicates diffusion-controlled processes, likely related to ionic transport through the coating and the oxide formed.

This evolution suggests that the coating initially acts as an effective barrier, but progressively allows electrolyte penetration, leading to interfacial reactions and the formation of corrosion products beneath the coating. The presence of multiple circuit elements reflects the coexistence of coating response, interfacial electrochemistry, and diffusion processes.

For the uncoated steel, the equivalent circuit evolves more rapidly, reflecting the obvious direct exposure of the substrate to the electrolyte. The absence of a protective coating results in lower resistance values and a more pronounced contribution of diffusion elements, associated with the formation of porous and non-protective corrosion layers.

Overall, the equivalent circuit analysis supports the EIS results, confirming that the polymeric coating enhances corrosion resistance by acting as a physical barrier while delaying the onset of electrochemical reactions at the metal surface.

The fitting procedure allowed the extraction of key electrochemical parameters. The resulting fitted values for representative immersion times are summarized in Table 3.

The fitted parameters confirm the time-dependent evolution of the electrochemical behavior observed in the impedance spectra. For the coated samples, a significant increase in coating resistance is observed from $1.35\times 10^{3}$ to $5.75\times 10^{3}$Ω·cm^2^ between 24 and 192 h, which is consistent with the increase in low-frequency impedance modulus and indicates enhanced barrier properties over time. This behavior is attributed to coating restructuring and the possible formation of protective interfacial species. Concurrently, the decrease in the constant phase exponent (*n*) suggests increasing interfacial heterogeneity, likely associated with water uptake and progressive electrolyte penetration.

At longer immersion times, the appearance of the Warburg element in the coated system indicates the onset of diffusion-controlled processes, confirming the transition from a purely capacitive barrier to a system influenced by mass transport. In contrast, the uncoated samples exhibit lower $R_{c}$ values and higher Warburg coefficients, reflecting the formation of porous and non-protective corrosion products and a stronger contribution of diffusion processes. Overall, these results support the superior corrosion resistance of the PVA/chitosan coating and are in good agreement with the qualitative interpretation of the EIS spectra.

## 4. Discussion

### 4.1. Structural Synergy and Intermolecular Interactions

The formulation of the 9.9:0.1 PVA/chitosan coating establishes a complex molecular architecture driven by the specific properties of its constituents. Spectroscopic analysis confirms that the bulk stability provided by PVA (Mw = 30,000 g/mol), characterized by its hydroxyl-rich backbone, is successfully integrated with the functional amino groups of chitosan. FTIR data reveals a broad absorption band between 3200–3500 cm^−1^, representing O–H stretching vibrations, while the presence of chitosan is substantiated by the amide I (C=O stretching) peak at 1650 cm^−1^ and the N–H bending vibrations of amino groups at 1550 cm^−1^.

Raman spectroscopy further demonstrates that these functional groups facilitate extensive intermolecular hydrogen bonding. This chemical cohesion confirms the development of a “cohesive polymer network” that functions as a high-density physical barrier. This structural synergy is the fundamental reason the system exhibits a purely capacitive response during early immersion; the density of the network effectively restricts ionic transport, thereby delaying the emergence of the Warburg (W_1_) diffusion element until the 192-h mark.

### 4.2. Structure–Function Relationship and Antimicrobial Potential of the Coating

Although the present study focuses on the electrochemical performance of the PVA/chitosan coating, its potential antimicrobial functionality is an important aspect for biomedical applications. The primary objective of this work was to characterize the electrochemical protection provided by the biopolymer blend; however, the presence of chitosan within the coating was confirmed through FTIR and Raman spectroscopy. In particular, the identification of characteristic bands corresponding to the amide I group (approximately 1650 cm^−1^) and N–H bending vibrations (around 1550 cm^−1^) indicates that the functional groups associated with chitosan are preserved in the final coating.

These groups are directly related to the polycationic nature of chitosan, which has been widely reported to exhibit antibacterial activity through electrostatic interactions with negatively charged microbial cell membranes, leading to increased membrane permeability and potential cell disruption. Therefore, the incorporation of chitosan into the PVA matrix suggests that the coating is chemically predisposed to provide not only corrosion protection but also potential antimicrobial functionality. This dual behavior is particularly relevant for surgical-grade steel instruments, where both corrosion resistance and infection control are critical. Nevertheless, microbiological assays were not conducted in this study, and future work will focus on evaluating the antibacterial performance of the coating under simulated physiological conditions.

### 4.3. Comparative Electrochemical Performance (Coated vs. Uncoated)

The Electrochemical Impedance Spectroscopy results characterize the performance of the protected system against the control across an immersion period extending to 216 h:

Solution Resistance Stability: In both systems, the solution resistance (R_1_) remained stable at high frequencies (10^2^–10^4^ Hz), confirming consistent electrolyte conditions within the Hanks’ Balanced Salt Solution.

Impedance Modulus Evolution: The coated samples demonstrate a significant enhancement in barrier efficacy. At the low-frequency limit (0.01 Hz), the impedance modulus (|Z|) increased from 1200–1400 Ω·cm^2^ at 24 h to a range of 5000–6000 Ω·cm^2^ by 216 h. This trend confirms the progression toward a more resistive interface, likely due to coating restructuring and the deposition of protective species.

Contrast with Uncoated Substrate: While the bare steel exhibits an increase in impedance over time, this is attributed to the formation of porous and non-protective corrosion product layers. Unlike the stable, dedicated barrier provided by the biopolymer, the uncoated system’s response is dominated by the rapid and irregular accumulation of surface oxides.

### 4.4. Mechanistic Evolution and the Warburg Phenomenon

The transition in equivalent circuit modeling confirms the progression from a purely capacitive barrier to a mass-transport-controlled regime.

During the early immersion phase (24 h), the system is modeled by a Randles circuit comprising solution resistance (R_1_), a constant phase element (Q_1_), and coating resistance (R_2_). Here, Q_1_ represents the non-ideal capacitive behavior of the biopolymer, which is associated specifically with surface heterogeneity and roughness. At this stage, R_2_ reflects a robust barrier that effectively limits ionic transport.

**Figure 6:**
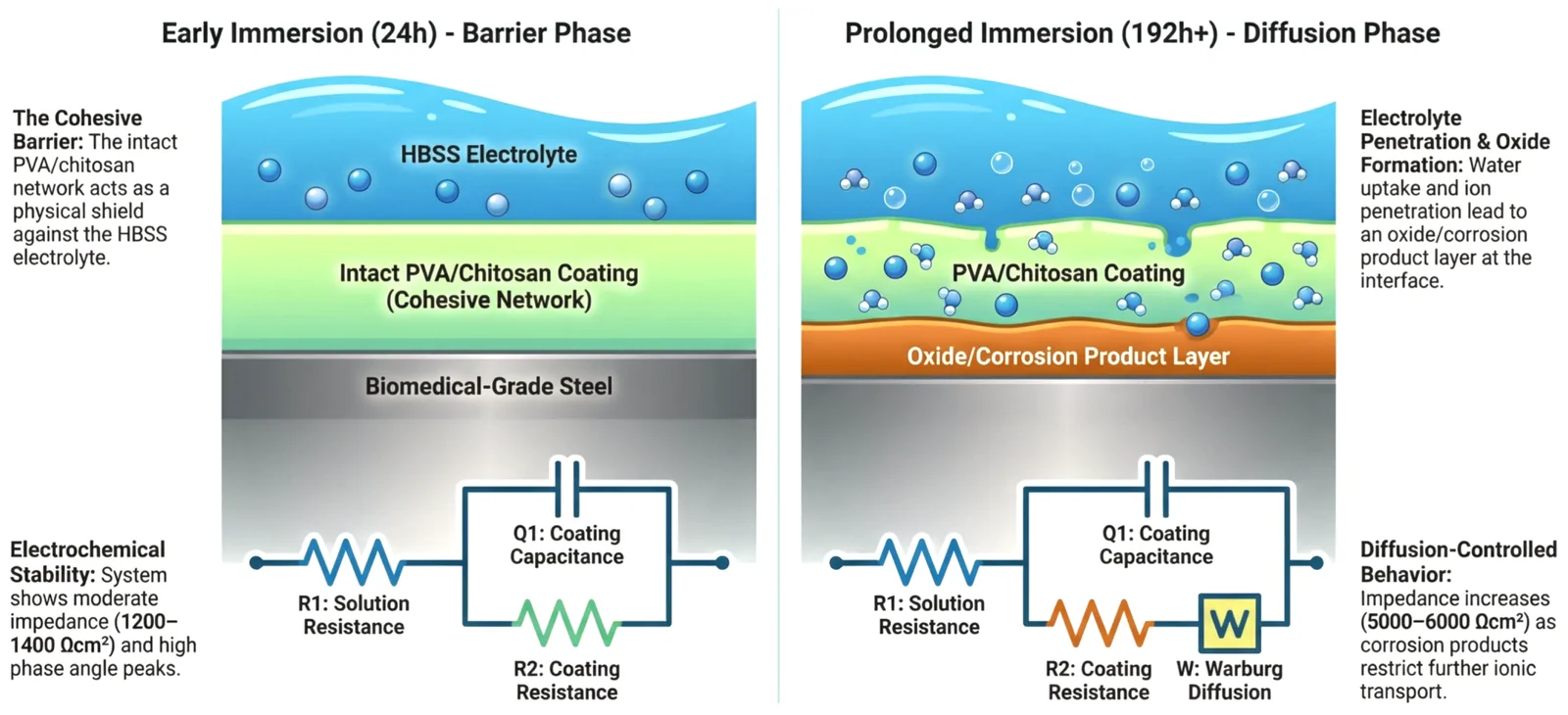
Mechanistic Evolution of PVA/Chitosan Anti-Corrosion Coating in HBSS Fluid.

By prolonged immersion (192 h), the model evolves into an extended circuit incorporating a Warburg element (W_1_). This transition is not merely indicative of diffusion but also signals the physical degradation of the once-cohesive polymer network. The phase angle response confirms this shift, showing a gradual decrease that indicates a significant deviation from ideal capacitive behavior. This evolution confirms that water uptake and electrolyte penetration eventually increase the heterogeneity at the coating/metal interface, necessitating the W_1_ element to account for the transport of ions through the coating and the underlying oxide layer. In Figure 6, a schematic representation of the time-dependent behavior of the PVA–chitosan coating onto biomedical steel in HBSS is proposed.

### 4.5. Implications for Biomedical Steel Protection

The metallic substrate consists of α-ferrite with a body-centered cubic structure. The analysis of the XRD data in Figure 1 reveals not only the primary ferrite peaks at 44.5°, 64.5°, and 82.1° but also secondary features such as FeCO_3_ (012) and (024). These peaks provide evidence of residual species and the initial surface condition of the steel prior to testing.

This ferritic microstructure is highly susceptible to localized corrosion when exposed to the chloride-rich environment of HBSS (pH 7.4). The PVA/chitosan coating serves as a critical physical shield that successfully delays the onset of electrochemical reactions. By maintaining high impedance and controlling the rate of mass transport, the coating effectively mitigates the release of metallic ions, which is vital for the long-term performance and biocompatibility of medical instruments.

## 5. Conclusions

The investigation of the time-dependent electrochemical behavior of the 9.9:0.1 PVA/chitosan coating system led to the following key conclusions:

The coating formulation successfully forms a cohesive and uniform film via dip-coating, maintaining structural integrity under simulated physiological conditions.

Effective corrosion protection is demonstrated by the significant increase in low-frequency impedance modulus, reaching values of 5000–6000 Ω·cm^2^ after 216 h of immersion, indicating enhanced barrier properties and interfacial stability.

The electrochemical response exhibits a clear mechanistic transition at prolonged immersion times (192 h), evolving from a predominantly barrier-controlled system, described by $R_{1}$, $Q_{1}$, and $R_{2}$, to a diffusion-influenced regime characterized by the appearance of the Warburg element ($W_{1}$).

The results confirm that PVA/chitosan biopolymer coatings provide a robust and time-dependent protective barrier for metallic substrates under physiological conditions (37 °C, pH 7.4).

Despite the onset of diffusion-controlled processes at extended immersion times, the coating maintains high impedance and effectively delays electrolyte penetration and interfacial degradation.

These findings highlight the potential of PVA/chitosan systems to enhance the electrochemical stability, durability, and biocompatibility of biomedical-grade steel, supporting their application in long-term clinical environments.

## Figures and Tables

**Table 1:** Comparison of representative biomedical coatings and their advantages and limitations relative to PVA/chitosan coating.

Coating	Advantages	Limitations	Applications
Hydroxyapatite (HA)	Bioactive; promotes osseointegration	Brittle; weak adhesion	Orthopedic, bone and dental implants [23]
Zirconia (ZrO_2_)	High strength; wear and corrosion resistant	Bioinert; aging effects	Dental implants [24]
TiN	Hard; corrosion resistant; low friction	Complex deposition; delamination	Surgical tools [26]
DLC	Very hard; low friction; wear resistant	Adhesion issues; high cost	Cardiovascular devices [25]
Silicon-based	Chemically stable; tunable properties	Lower strength; limited clinical use	Experimental biomedical coatings [27]
PVA/Chitosan	Biocompatible; biodegradable; antibacterial potential; low cost	Lower strength; swelling in aqueous media	Surgical steel protection [16]

**Table 2:** Composition of Hanks’ Balanced Salt Solution (HBSS) used as electrolyte.

Component	Concentration (g/L)	Molarity (mM)
NaCl	8.00	137
KCl	0.40	5.4
CaCl_2_	0.14	1.3
NaHCO_3_	0.35	4.2
KH_2_PO_4_	0.06	0.44
Na_2_HPO_4_·2H_2_O	0.06	0.34
MgSO_4_·7H_2_O	0.10	0.41
MgCl_2_·6H_2_O	0.10	0.49
Glucose	1.00	5.5

**Table 3:** Equivalent electrical circuit (EEC) parameters obtained from fitting of EIS spectra.

Sample	Time (h)	$R_{1}$ (Ω·cm^2^)	$R_{2}$ (Ω·cm^2^)	$Q_{1}$ (S·s^*n*^·cm^−2^)	*n*	*W* (Ω·s^−1/2^)
Coated	24	18.5	$1.35\times 10^{3}$	$3.2\times 10^{-5}$	0.92	–
Coated	192	19.2	$5.75\times 10^{3}$	$1.1\times 10^{-5}$	0.81	210
Bare	24	17.9	$8.5\times 10^{2}$	$5.6\times 10^{-5}$	0.88	150
Bare	192	18.7	$1.6\times 10^{3}$	$2.9\times 10^{-5}$	0.76	320

## Data Availability

The original data presented in the study are openly available in the Zenodo repository at https://doi.org/10.5281/zenodo.19376219.

## Abbreviations

The following abbreviations are used in this manuscript:

PVA	Poly(vinyl alcohol)
HBSS	Hanks’ Balanced Salt Solution
EIS	Electrochemical Impedance Spectroscopy
FTIR	Fourier Transform Infrared Spectroscopy
ATR	Attenuated Total Reflectance
XRD	X-ray Diffraction
OCP	Open Circuit Potential
CPE	Constant Phase Element
bcc	Body-centered cubic
